# Draft Genome Sequences and Annotation of *Enterococcus faecium* Strain LCT-EF20

**DOI:** 10.1128/genomeA.00083-12

**Published:** 2013-01-24

**Authors:** De Chang, Yuanfang Zhu, Jiapeng Chen, Xiangqun Fang, Tianzhi Li, Junfeng Wang, Yinghua Guo, Longxiang Su, Guogang Xu, Yajuan Wang, Zhenhong Chen, Changting Liu

**Affiliations:** a Nanlou Respiratory Diseases Department, Chinese PLA General Hospital, Beijing, China; b BGI-Shenzhen, Shenzhen, People’s Republic of China

## Abstract

The space environment is reported to cause biological alterations in microorganisms, such as growth, drug resistance, and virulence. Here, we present the model of *Enterococcus faecium* to investigate the effects of space conditions on the microbe and on the whole-genome sequences of the strain LCT-EF20 after being exposed to space flight.

## GENOME ANNOUNCEMENT

Previous studies have suggested that space flight may alter the effectiveness of antibiotics against microbes (1, 2). The inhibition of bacterial growth in space requires higher concentrations of various drugs due to the reduced efficacy of the drugs and increased bacterial resistance (3, 4). In addition, bacteria exposed to space flight displayed increased virulence as discovered through murine infection assays (5, 6). *Enterococcus faecium* is a ubiquitous opportunistic pathogen grown in human and animal intestines (7). Since *E. faecium* coexists in human intestines, it is necessary to assess the behavior and genome of *E. faecium* under space flight conditions. Therefore, in order to prevent astronauts from infectious disease in space and to provide insight into the identification of candidate targets for refractory infections on the ground, the genome of *E. faecium* (LCT-EF20) under space flight conditions was sequenced and analyzed.

The clustering of shotgun reads and manual finishing of the assembly resulted in 111 contiguous sequences (contigs) with an N_50_ of 73,148 bp and 120× average genome coverage. The combined contig length was 2,718,930 bp, connected to 34 scaffolds (>500 bp each), with total length of 2,809,068 bp containing 90,138-bp gap regions. The *G+C* content of the overall assembly was 38%. The assembly contains 2,807 genes with a length of 2,431,857 bp consisting of 86.57% in the genome. The transposon sequences were predicted using RepeatMasker and RepeatProteinMasker software programs, and tandem repeat sequences were predicted using Tandem Repeats Finder (TRF) (8). Finally, 313-kb different transposable element (TE)-related sequences consisting of 0.44% in the genome were found. Protein-coding sequences were retrieved from the chromosomes of *E. faecium* strain LCT-EF20.

The gene functions were annotated using BLASTP to identify all coding protein sequences comparing the Kyoto Encyclopedia of Genes and Genomes (KEGG) (9), Clusters of Orthologous Groups (COG), Swiss-Prot, TrEMBL, Gene Ontology (GO), and nonredundant (NR) databases. Homologous proteins were identified by BLASTP with the criteria of an *e* value cutoff of 1*e−*5 and a minimum aligned sequence length coverage of 50% of a query sequence. Similarly, using the above criteria, 1,896 protein families were yielded, with 1,885 single-copy protein families. Genome island prediction, prophage sequences, and clustered regularly interspaced short palindromic repeats (CRISPRs) were also predicted separately, but no sequence was found.

### Nucleotide sequence accession number.

This whole genome sequence of *E. faecium* LCT-EF20 has been deposited at DDBJ/EMBL/GenBank under the accession no. ANAI00000000.
